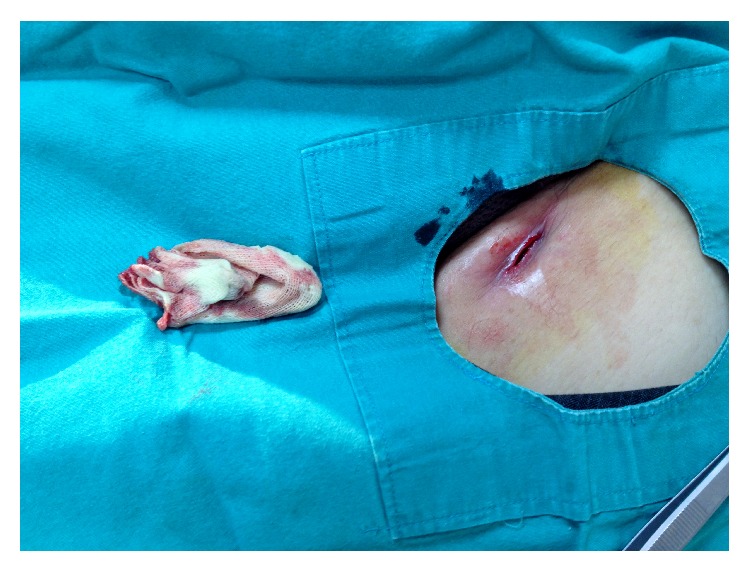# An Extremely Rare Complication of Varicose Vein Surgery: Retained Foreign Body

**DOI:** 10.1155/2014/584580

**Published:** 2014-11-09

**Authors:** Orhan Fındık, Ufuk Aydın, Çağrı Düzyol, Özgür Barış, Cevdet Uğur Koçoğulları

**Affiliations:** Department of Vascular and Cardiac Surgery, Derince Research and Educational Hospital, 41000 Kocaeli, Turkey

## Abstract

Foreign body is among complications of surgery. But as a complication of varicose vein surgery it was reported extremely rarely and, to our knowledge, there is only one paper in the literature. A case with retained sponge which was detected five months after varicose vein surgery was presented.

## 1. Introduction

Abdominal operations are the number one causes of the retained foreign bodies such as sponge, instruments which are followed by operations of chest, pelvis, breast, cranium, and extremities. Although true incidence of retained foreign body is not known, its frequency in operations for both abdominal and pelvic regions and only for abdominal region is 1/100–5000 and 1/1000–1500, respectively [1–3]. Every year more than 1500 cases of retained foreign bodies are reported in United States [4].

Varicose vein surgeries are commonly performed for chronic venous insufficiency and as far as we know there was only one paper reporting retained foreign body after varicose vein surgery in the literature [5].

## 2. Case

**Figure 1:**
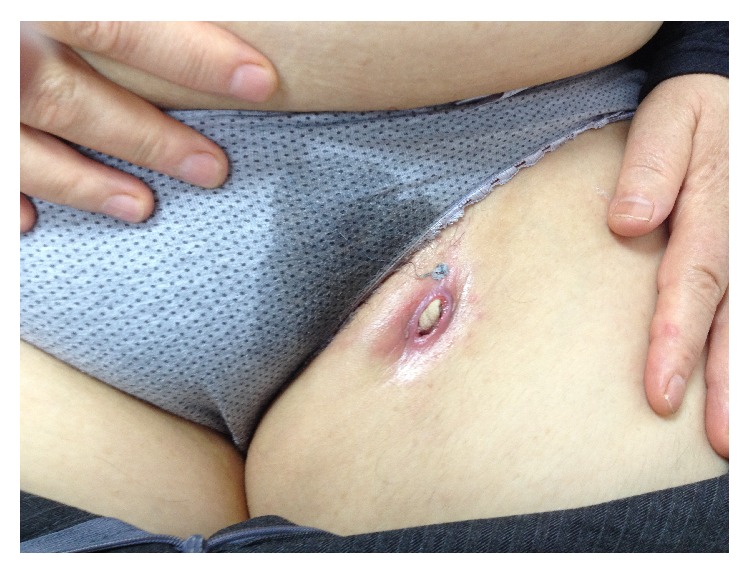


A 55-year-old female admitted with complaints of pain and swelling of left inguinal region. It was learnt that she had been underwent varicose vein surgery at a different medical centre five months before. After all, she noticed swelling, pain, discharge, and redness over incision site on left inguinal region and was treated with a different series of antibiotics for 4 months. At her presentation to our clinic, swelling of left groin was noticed without any discharge (Figure 1). We were suspected of foreign body in this patient so superficial ultrasonography was performed. And also complete blood count, erythrocyte sedimentation rate, and CRP were ordered. The blood results were normal. Ultrasonography revealed a mass with a leafy appearance suggesting foreign body. And incision was opened and a tip of a sterile sponge was seen and taken out (Figure 2). After two specimens were taken from bottom of incision for culture, the lesion was cleared. Since culture results were negative, no antibiotics were prescribed. On control day, two weeks later, the incision was completely normal.

## 3. Discussion

There is always a probability to forget sponge or instruments after minor or major surgeries. But the diagnosis is not so easy. And the diagnosis could be made immediately or years after operation. The symptoms may be local such as swelling, pain, discharge, and abscess and/or systemic such as fever, nausea, and vomiting. Sometimes the patients may not complain of any symptom [6].

Abdomen, thorax, and pelvis are the most common and most risky anatomic sites for the complication. Nevertheless, small anatomic sites such as ethmoidal sinus and paraspinal spaces are also dangerous for retained foreign body. Common risk factors for the retained foreign body are emergency operations, abdominal, thoracic, and pelvic operations, operations during which strategy had to be changed, and patients with a high body mass index. To decrease the frequency of the complication, the surgical team should be experienced and careful and should count the materials and instruments, such as sterile sponge, and when there is a suspicion for retained foreign body, radiography must be taken before the end of surgery. Direct radiography, ultrasonography, computed tomography, and magnetic resonance with knowledge of recent surgery are important diagnostic methods for the detection of the retained foreign body [7].

Mostly because of possible legal reasons, reports of the foreign bodies were published rarely both in our country and in other countries. Nowadays, the most common operation in cardiovascular field is varicose vein surgery for chronic venous insufficiency. And varicose vein surgery is usually planned operations and made electively. Thus, limited size of incision, small anatomical spaces, and its elective nature of the varicose surgery are the key factors to be a rare cause for retained foreign body.

Although the varicose vein surgery is one of the very rare causes of retained foreign body, it must be kept in mind that careful counting of sponges used for the bleeding control especially in obese patients who underwent stripping procedure and final observation of the incision site before closure are recommended to prevent the complication.

## Figures and Tables

**Figure 2:**